# Mutations in *CCDC39* and *CCDC40* are a major cause of primary ciliary dyskinesia with microtubule disorganisation

**DOI:** 10.1186/2046-2530-1-S1-P1

**Published:** 2012-11-16

**Authors:** D Antony, A Becker-Heck, M Forouhan, M Schmidts, A Onoufriadis, A Shoemark, M Dixon, C Jackson, P Goggin, H Olbrich, C O’Callaghan, J Lucas, C Hogg, EMK Chung, H Omran, HM Mitchison

**Affiliations:** 1 Molecular Medicine Unit, University College London (UCL) Institute of Child Health, UK; 2 Department of Pediatrics and Adolescent Medicine, University Hospital Muenster, Germany; 3 Department of Paediatric Respiratory Medicine, Royal Brompton and Harefield NHS Trust, UK; 4 Primary Ciliary Dyskinesia Group, University of Southampton Faculty of Medicine, Southampton NIHR Respiratory Biomedical Research Unit, Southampton General Hospital, UK; 5 University of Leicester and Leicester Royal Infirmary, UK; 6 General and Adolescent Paediatric Unit, UCL Institute of Child Health, UK

## 

Primary ciliary dyskinesia (PCD) is a genetically heterogeneous inherited disorder characterised by recurrent respiratory tract infections, bronchiectasis and subfertility which arises from cilia/sperm dysmotility associated with axonemal ultrastructural abnormalities. Laterality is randomized with ~50% of patients having *situs inversus*. Up to 15% of PCD cases show perturbation of the 9+2 microtubule structure and loss of the inner dynein arms, and these have tended to be referred to as ‘radial spoke defect’ cases. The radial spokes are essential for axoneme motility, mediating signal transduction between the central microtubular pair and dynein arm motors. Two genes causing this specific ultrastructural defect are known: *CCDC39* (Merveille et. al., Nat Genet. 2011 43:72-8) and *CCDC40* (Becker-Heck et. al. Nat Genet. 2011 43:79-84). We sequenced these genes in 22 PCD families with an ultrastructural defect involving microtubule disorganisation, either with or without accompanying loss of the inner dynein arms. We found recesively inherited *CCDC39* mutations in 8/22 families and *CCDC40* mutations in 7/22 families in the cohort, jointly accounting for a remarkable 68% (15/22) of families. The majority of *CCDC39* and *CCDC40* mutations were nonsense or frameshift resulting in early protein truncation, predicted to cause major disruption to the axoneme. Furthermore, there was a preponderance of homozygous mutations accounting for disease, even in families from outbred populations. Our results highlight the key role of the *CCDC39* and *CCDC40* genes in PCD with radial spoke defect, and suggest that disease is associated with complete protein loss (null alleles). These two genes represent prime targets for genetic testing in this disease phenotype. Work is in progress to identify the disease genes in the remaining patients within this subgroup, by next generation whole exome sequencing.

